# Supplementary material for: The Mouse Inferior Colliculus Responds Preferentially to Non-Ultrasonic Vocalizations
Source: eNeuro. 2024 Apr 10;11(4):ENEURO.0097-24.2024. doi: 10.1523/ENEURO.0097-24.2024 (PMC11015948; doi:10.1523/ENEURO.0097-24.2024)
Supplement: Table 3-1 — Statistical comparisons of the distributions of upper frequency limits among IC units by CF group and subdivision. All p-values were derived using independent-samples Kruskal-Wallis tests and adjusted using Benjamini-Hochberg corrections. Significance level = 0.05; False Discovery Rate = 0.05. Statistically significant values are shown with an asterisk. Raw p-values were not adjusted if the null hypothesis (no difference between medians) was not rejected. For all analyses with adjusted p values, degrees of freedom = 2. AllCF = all CF groups combined; AllSubs = all subdivisions combined; n.s.=non-significant. Download Table 3-1, PDF file. [file eneuro-11-ENEURO.0097-24.2024-s001.pdf]

Table 3-1

Group Comparisons	Median (kHz)	Interquartile Range (Q_3-Q_1)	Independent Sample Kruskal-Wallis p-value	BH Critical p-value
CIC, 10 vs. 20 kHz	29, 34	33.67, 37.46	n.s.=0.07	0.04
CIC, 10 vs. 40 kHz	29, 64	33.67, 65.18	<0.001*	0.002
CIC, 20 vs. 40 kHz	34, 64	37.46, 65.18	<0.001*	0.005
DCIC, 10 vs. 20 kHz	46, 48.5	46.98, 51.32	n.s.=0.15	0.05
DCIC, 10 vs. 40 kHz	46, 76	46.98, 70.19	<0.001*	0.01
DCIC, 20 vs. 40 kHz	48.5, 76	51.32, 70.19	<0.001*	0.01
ECIC, 10 vs. 20 kHz	29, 40	32.62, 46.1	<0.001*	0.02
ECIC, 10 vs. 40 kHz	29, 63	32.62, 61.26	<0.001*	0.03
ECIC, 20 vs. 40 kHz	40, 63	46.1, 61.26	0.003*	0.04
AllSubs, 10 vs. 20 kHz	--	--	<0.001*	0.02
AllSubs, 10 vs. 40 kHz	--	--	<0.001*	0.007
AllSubs, 20 vs. 40 kHz	--	--	<0.001*	0.01
CIC vs. DCIC, 10 kHz	29, 46	33.67, 46.98	<0.001*	0.03
ECIC vs. DCIC, 10 kHz	29, 46	32.62, 46.98	<0.001*	0.04
ECIC vs. CIC, 10 kHz	29, 29	32.62, 33.67	n.s.=0.64	0.05
CIC vs. DCIC, 20 kHz	34, 48.5	37.46, 51.32	<0.001*	0.03
ECIC vs. DCIC, 20 kHz	40, 48.5	46.1, 51.32	0.03*	0.04
ECIC vs. CIC, 20 kHz	40, 34	46.1, 37.46	<0.001*	0.03
AllSubs, 40kHz	--	--	0.09	unadjusted
CIC vs. DCIC, AllCF	--	--	0.001*	0.03
ECIC vs. DCIC, AllCF	--	--	<0.001*	0.02
ECIC vs. CIC, AllCF	--	--	0.003*	0.04

Statistical comparisons of the distributions of upper frequency limits among IC units by CF group and subdivision. All p-values were derived using independent-samples Kruskal-Wallis tests and adjusted using Benjamini-Hochberg corrections. Significance level = 0.05; False Discovery Rate= 0.05. Statistically significant values are shown with an asterisk. Raw p-values were not adjusted if the null hypothesis (no difference between medians) was not rejected. For all analyses with adjusted p values, degrees of freedom = 2. AllCF = all CF groups combined; AllSubs = all subdivisions combined; n.s.=non-significant.
