# Supplementary material for: The Mouse Inferior Colliculus Responds Preferentially to Non-Ultrasonic Vocalizations
Source: eNeuro. 2024 Apr 10;11(4):ENEURO.0097-24.2024. doi: 10.1523/ENEURO.0097-24.2024 (PMC11015948; doi:10.1523/ENEURO.0097-24.2024)
Supplement: Table 3-2 — Statistical comparisons of the distributions of upper frequency limits among CIC units by CF, sex and estrous stage. All p-values were derived using independent-samples Kruskal-Wallis tests and adjusted using Benjamini-Hochberg corrections. Significance level = 0.05; False Discovery Rate = 0.05. Statistically significant values are shown with an asterisk. Raw p-values were not adjusted if the null hypothesis (no difference between medians) was not rejected. For all analyses with adjusted p values, degrees of freedom = 2. Fnonest. = non-estrus female; Fest. = estrus female; M = Male; n.s.=non-significant. Download Table 3-2, PDF file. [file eneuro-11-ENEURO.0097-24.2024-s002.pdf]

Table 3-2

Group Comparisons	Median (kHz)	Interquartile Range (Q_3-Q_1)	Independent Sample Kruskal-Wallis p-value	BH Critical p-value
$F_{nonest.}$, 10 vs. 20 kHz	19, 37	31.26, 38.54	n.s.=0.08	0.05
$F_{nonest.}$, 10 vs. 40 kHz	19, 64	31.26, 64.1	<0.001*	0.02
$F_{nonest.}$, 20 vs. 40 kHz	37, 64	38.54, 64.1	<0.001*	0.02
$F_{est.}$, 10 vs. 20 kHz	36, 56	36.62, 53.2	0.002*	0.03
$F_{est.}$, 10 vs. 40 kHz	36, 61	36.62, 60	0.004*	0.03
$F_{est.}$, 20 vs. 40 kHz	56, 61	53.2, 60	n.s.=0.35	0.04
M, 10 vs. 20 kHz	29, 31	33.76, 34.55	n.s.=0.61	0.05
M, 10 vs. 40 kHz	29, 65	33.76, 65.95	<0.001*	<0.001
M, 20 vs. 40 kHz	31, 65	34.55, 65.95	<0.001*	0.01
AllSex, 10 vs. 20 kHz	--	--	n.s.=0.07	0.04
AllSex, 10 vs. 40 kHz	--	--	<0.001*	0.01
AllSex, 20 vs. 40 kHz	--	--	<0.001*	0.01
M vs. $F_{nonest.}$, 20 kHz	31, 37	34.55, 38.54	0.03*	0.04
M vs. $F_{est.}$, 20 kHz	31, 56	34.55, 53.2	<0.001*	0.02
$F_{nonest.}$ vs. $F_{est.}$, 20kHz	37, 56	38.54, 53.2	<0.001*	0.03
AllSex, 10kHz	--	--	0.33	unadjusted
AllSex, 40kHz	--	--	0.23	unadjusted

Statistical comparisons of the distributions of upper frequency limits among CIC units by CF, sex and estrous stage. All p-values were derived using independent-samples Kruskal-Wallis tests and adjusted using Benjamini-Hochberg corrections. Significance level = 0.05; False Discovery Rate= 0.05. Statistically significant values are shown with an asterisk. Raw p-values were not adjusted if the null hypothesis (no difference between medians) was not rejected. For all analyses with adjusted p values, degrees of freedom = 2. $F_{nonest.}$ = non-estrus female; $F_{est.}$ = estrus female; M = Male; n.s.=non-significant.
