# Supplementary material for: The Mouse Inferior Colliculus Responds Preferentially to Non-Ultrasonic Vocalizations
Source: eNeuro. 2024 Apr 10;11(4):ENEURO.0097-24.2024. doi: 10.1523/ENEURO.0097-24.2024 (PMC11015948; doi:10.1523/ENEURO.0097-24.2024)
Supplement: Figure 5-1 — Responses of IC population to WAV stimuli as function of CF. A-F. Population response to the WAV stimulus indicated in the sonogram. Each plot shows excitatory (red) and inhibitory (blue) SDF responses to stimuli at 60 dB SPL peak. See Figure 5 for protocol. Each plot shows the same 944 units as in Figures 3A and 5, the subset of 1212 sound-responsive IC units that responded to at least one of the 60 dB SPL WAV stimuli. Stimulus spectrograms and number of responding units shown above main plot. Download Figure 5-1, PDF file. [file eneuro-11-ENEURO.0097-24.2024-s003.pdf]

FIGURE 5-1

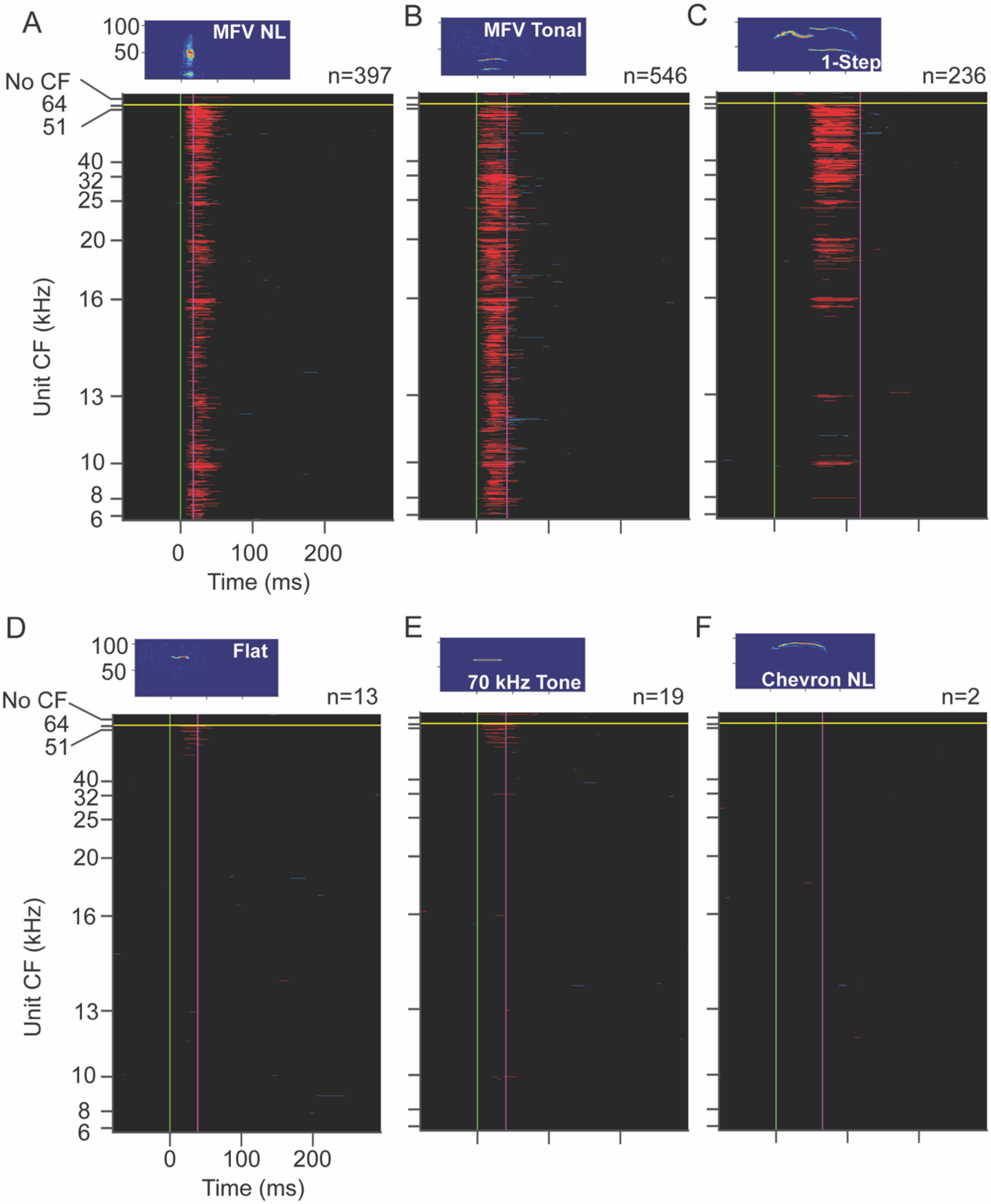

Figure 5-1. Responses of IC population to WAV stimuli as function of CF. A-F. Population response to the WAV stimulus indicated in the sonogram. Each plot shows excitatory (red) and inhibitory (blue) SDF responses to stimuli at 60 dB SPL peak. See Figure 5 for protocol. Each plot shows the same 944 units as in Figures 3A and 5, the subset of 1212 sound-responsive IC units that responded to at least one of the 60 dB SPL WAV stimuli. Stimulus spectrograms and number of responding units shown above main plot.
