# Supplementary material for: The Mouse Inferior Colliculus Responds Preferentially to Non-Ultrasonic Vocalizations
Source: eNeuro. 2024 Apr 10;11(4):ENEURO.0097-24.2024. doi: 10.1523/ENEURO.0097-24.2024 (PMC11015948; doi:10.1523/ENEURO.0097-24.2024)
Supplement: Table 9-1 — Statistical analyses of IC subdivision responsiveness to categories of social vocalizations. All p-values were derived using a Linear Mixed Effects Model and adjusted using Benjamini-Hochberg corrections. Significance level = 0.05; False Discovery Rate = 0.05. Statistically significant values are shown with an asterisk. There were no sex differences among CIC units in their representation of vocal categories, thus the p-value (0.09) was not adjusted. Vocal Cat = vocal category; AllSubs = all subdivisions combined; n.s.=non-significant. Download Table 9-1, PDF file. [file eneuro-11-ENEURO.0097-24.2024-s004.pdf]

Table 9-1

Group Comparisons	Independent Sample Linear Mixed Effects Model p-value	BH Critical p-value
CIC, Vocal Cat	<0.001*	0.003
CIC, Vocal Cat 1 vs. 2	<0.001*	0.006
CIC, Vocal Cat 1 vs. 3	<0.001*	0.008
CIC, Vocal Cat 2 vs. 3	<0.001*	0.01
DCIC, Vocal Cat	<0.001*	0.01
DCIC, Vocal Cat 1 vs. 2	<0.001*	0.02
DCIC, Vocal Cat 1 vs. 3	<0.001*	0.02
DCIC, Vocal Cat 2 vs. 3	<0.001*	0.02
ECIC, Vocal Cat	<0.001*	0.03
ECIC, Vocal Cat 1 vs. 2	<0.001*	0.03
ECIC, Vocal Cat 1 vs. 3	<0.001*	0.03
ECIC, Vocal Cat 2 vs. 3	<0.001*	0.03
Vocal Cat 1, AllSubs	n.s.=0.06	0.04
Vocal Cat 2, AllSubs	<0.001*	0.04
Vocal Cat 2, CIC vs. ECIC	<0.001*	0.04
Vocal Cat 2, CIC vs. DCIC	0.002*	0.04
Vocal Cat 2, DCIC vs. ECIC	n.s.=0.91	0.05
Vocal Cat 3, AllSubs	n.s.=0.4	0.05
CIC, Sex	0.09	unadjusted

Statistical analyses of IC subdivision responsiveness to categories of social vocalizations. All p-values were derived using a Linear Mixed Effects Model and adjusted using Benjamini-Hochberg corrections. Significance level = 0.05; False Discovery Rate= 0.05. Statistically significant values are shown with an asterisk. There were no sex differences among CIC units in their representation of vocal categories, thus the p-value (0.09) was not adjusted. Vocal Cat = vocal category; AllSubs = all subdivisions combined; n.s.=non-significant.
