# Supplementary material for: The Mouse Inferior Colliculus Responds Preferentially to Non-Ultrasonic Vocalizations
Source: eNeuro. 2024 Apr 10;11(4):ENEURO.0097-24.2024. doi: 10.1523/ENEURO.0097-24.2024 (PMC11015948; doi:10.1523/ENEURO.0097-24.2024)
Supplement: Figure 11-1 — Responses of IC units from UNANEST mice to WAV stimuli as function of CF. A-F. Population response to the WAV stimulus indicated in the sonogram. Each plot shows excitatory (red) and inhibitory (blue) SDF responses to stimuli at 60 dB SPL peak. Each horizontal line represents response of one unit. See Figure 5 for protocol. Each plot shows the same 261 units as in Figures 10C and 11, the subset of sound-responsive IC units from UNANEST animals that responded to at least one of the 60 dB SPL WAV stimuli. Download Figure 11-1, PDF file. [file eneuro-11-ENEURO.0097-24.2024-s005.pdf]

FIGURE 11-1

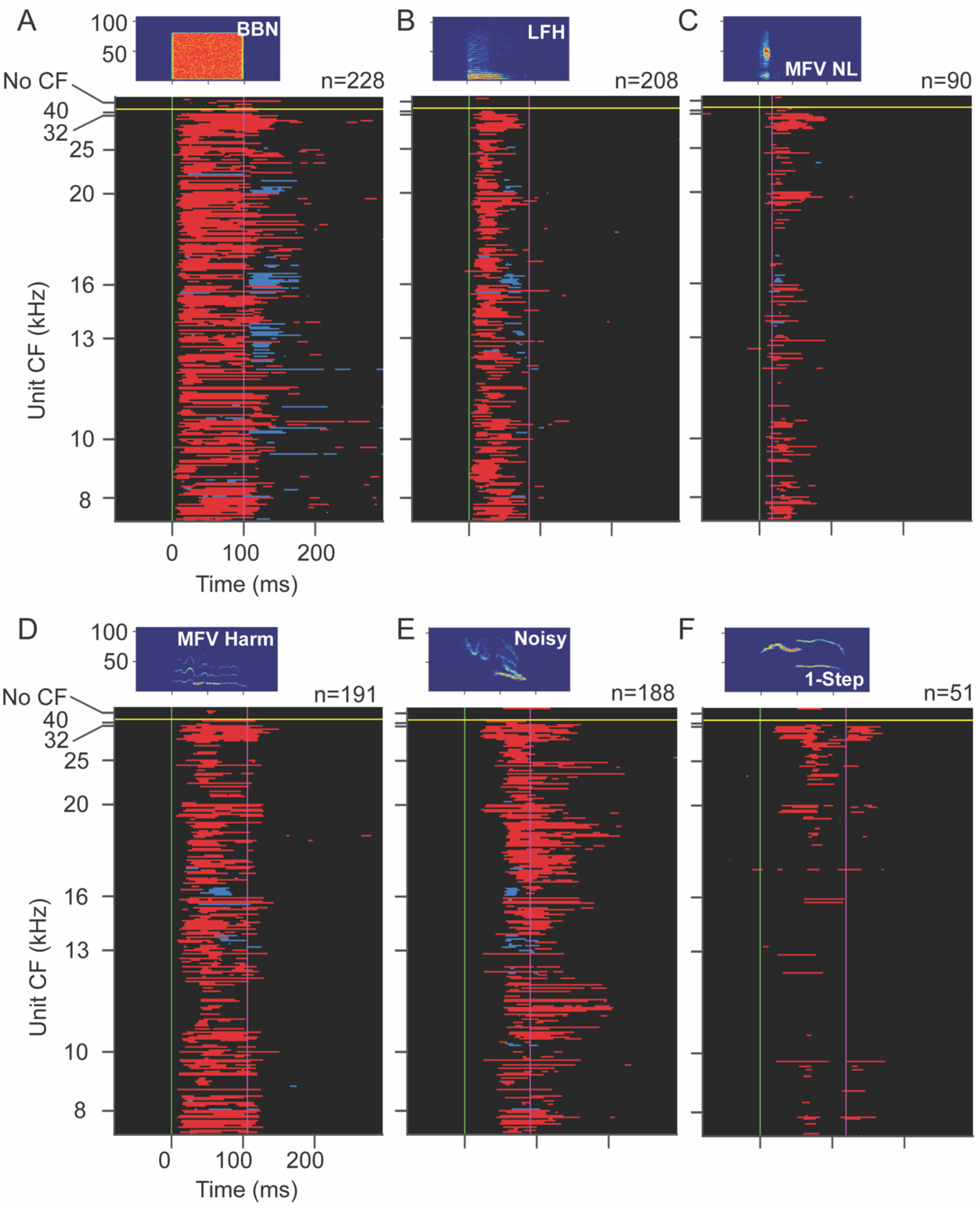

Figure 11-1. Responses of IC units from UNANEST mice to WAV stimuli as function of CF. A-F. Population response to the WAV stimulus indicated in the sonogram. Each plot shows excitatory (red) and inhibitory (blue) SDF responses to stimuli at 60 dB SPL peak. Each horizontal line represents response of one unit. See Figure 5 for protocol. Each plot shows the same 261 units as in Figures 10C and 11, the subset of sound-responsive IC units from UNANEST animals that responded to at least one of the 60 dB SPL WAV stimuli.
